# The impact of volunteer service on moral education performance and mental health of college students

**DOI:** 10.1371/journal.pone.0294586

**Published:** 2024-04-16

**Authors:** Zhiwei Lv, Changtian Ying, Jiayi Chen

**Affiliations:** 1 School of Education, Central China Normal University, Wuhan, Hubei, 430079, China; 2 School of Foreign Languages, Shaoxing University, Shaoxing, Zhejiang, 312000, China; 3 School of Mechanical and Electrical Engineering, Shaoxing University, Shaoxing, Zhejiang, 312000, China; 4 Youth League Committee, Shaoxing University, Shaoxing, Zhejiang, 312000, China; University of Maribor, SLOVENIA

## Abstract

**Background:**

Moral education in colleges and universities is an important part of the talent training system, including moral education curriculum, moral education practice, mental health education. Volunteer service is a public welfare act in which volunteers volunteer their time, knowledge, property, technology, with the ultimate goal of helping others and serving the society without personal compensation. As an innovative form of moral education practice in colleges and universities, college students’ voluntary service is of great significance in promoting the reform and innovation of moral education, enhancing the affinity, appeal and influence of moral education, and building a positive psychology for college students.

**Subjects and methods:**

As an effective carrier of moral education practice in colleges and universities, voluntary service is helpful to enhance the effectiveness of moral education practice and construct the positive psychology of college students. This project is based on the actual situation of college students participating in volunteer services, and collected the volunteer services of 4545 college students in Zhejiang Province. Through model construction and data modeling, the correlation between college students’ participation in volunteer service and their moral education performance and mental health was analyzed, and the basic path and guarantee measures to promote the role of volunteer service in moral education and positive psychological construction were deeply explored.

**Results:**

From the correlation analysis of students’ voluntary service participation, moral education performance and voluntary service motivation, students’ attributes are determined according to their voluntary service participation, so as to predict their moral education performance and mental health level.

**Conclusion:**

College students’ voluntary service is partially positively related to their moral education performance and mental health. In order to improve students’ moral education performance and mental health, we can optimize the participation frequency, participation duration, participation ways and type structure of voluntary service, constantly increase the participation frequency of voluntary service, increase the duration of voluntary service, broaden the participation ways of voluntary activities, and enrich the types of voluntary service activities.

## 1. Moral function of college students’ volunteer service

The college students’ volunteer service activities in the process of its development comprehensively use a variety of moral education methods, which has played a positive role in the formation of good ideological and moral quality of college students, playing a unique role. The in-depth analysis and research of its moral education function is an important prerequisite and basis for us to further enhance its education and play the role of moral education in colleges and universities.

### 1.1 The guiding function of moral cultivation

college students’ volunteer service has an important guiding function in college moral education. It can guide college students’ thoughts, feelings and behaviors, making them more in line with the needs of social development. Volunteer service activity is a public welfare undertaking in which the public participate. College student volunteers go to the grass-roots level, participate in public welfare work, provide services for others and the society, observe and understand the society intuitively, participate in improving the living conditions of vulnerable groups, resolving social public crisis and other social public welfare events, and help to increase social awareness, realize the unity of self cognition and cognitive society [1]. Volunteer service activities are warm, which convey warmth and care, and have a positive guiding role in people’s feelings. In the process of volunteer service, college student volunteers feel their self-worth, experience sharing and harvest, and build a harmonious interpersonal relationship. Volunteer service can also help guide university volunteers to choose an active and healthy lifestyle, form a benevolent behavior habit, and thus realize the awakening of individual behavior [2].

### 1.2 The coordinating function of social harmony

The development of college students’ volunteer service is usually organized. College students must join a certain volunteer service organization to have the opportunity and platform to carry out volunteer service work, which also enables individual students to have a certain organizational ownership. In order to achieve the long-term sustainable development of volunteer service projects, when personal interests and group interests conflict, college student volunteers usually learn to coordinate the development between individuals and groups through positive thinking and self adjustment, and promote the maximization of group interests and the maximum realization of group goals through individual efforts [3]. They also perceive individual values, find themselves, and improve themselves in group life. College student volunteers also need to coordinate the relationship with their clients. College student volunteers have not gone out of school yet, and they have a relatively narrow understanding of the society and little social experience. They meet a variety of people in volunteer service activities, gradually understand the society, and also experience the life of people with different occupations, incomes, and life circles. In this process, they learn to be tolerant and receptive to their service objects, and establish a harmonious relationship between people living in harmony.

### 1.3 The constructive function of positive psychology

The cultivation of positive psychology is a process of psychological formation, which follows the general law of psychological formation of "knowing, feeling and meaning" [4]. Among them, positive emotional experience is the greatest gain for college students to participate in social work services. B. L. Frederick proposed that "some discrete positive emotions have the ability to extend people’s instantaneous knowledge and behavior, and can build and enhance people’s personal physical strength, intelligence, social coordination and other resources. Positive emotions can reduce negative emotions" [5]. The process of helping service objects to solve their life difficulties and their sense of achievement after achievements; The sense of life happiness obtained by comparing their own experience with the plight of the service object; The indomitable life attitude and indomitable spirit of the vulnerable groups get a sense of progress; The pleasure of gaining knowledge through personal growth; The expansion of interpersonal circle, the excitement of unity and cooperation among teammates and other positive emotions promote the overall development of college students’ individual body and mind. In addition. College students consciously participate in social work services, can overcome difficulties and setbacks in the service process, and show good will qualities such as self-awareness, decisiveness and tenacity. College students independently choose corresponding service activities, consciously complete the whole process from planning, implementation to summary, and make arbitrary judgments, thoughts and implementation decisions. A large amount of coordination work before and after planning and preparation, as well as emergencies in the implementation process, require college students to keep calm and respond in a timely manner. In the face of unfamiliar service objects, unknown fields and complex work, college students are given negative emotions. Through overcoming anxiety, fear and timidity, they have formed a positive psychological state of perseverance.

### 1.4 The motivation function of self growth

College student volunteers practice the spirit of volunteerism in the actual work of voluntary activities, experience the joy of dedication in practice, walk with examples, and move with each other, encouraging them to constantly improve their own ideas, strengthen personal ethics, shine youth in the passion of struggle, and strengthen their life ideals in selfless dedication. Volunteer service has imperceptibly changed college students’ understanding of the world, life, values, honor and disgrace, played a realistic role in encouraging college students’ ideas, and helped them gradually become a person with ideological progress and all-round development in the process of self-development [6–12]. In social life, individuals can only contribute their wisdom and ability to the society, use their own strength to promote social civilization and progress, and obtain social respect and recognition. College student volunteers have contributed their knowledge and strength to the whole society, and achieved the transcendence from the "small self" to the "big self" [6]. While enhancing the enthusiasm of college students to participate in volunteer service activities, we should also pay attention to the incentive role of example demonstration, attract more students to participate in the volunteer service team, learn from and approach the example.

## 2. An analysis of the current situation of college students’ volunteer service

In order to deeply study the current situation and existing problems of college students’ volunteer service, the questionnaire "college students’ Participation in Volunteer Service" (see supporting file) was designed to conduct a questionnaire survey on the effect of volunteer service in colleges and universities in Zhejiang Province on moral education practice. The ethical review of this study was approved by the Ethics Committee for the Study of Ideological and Political Education for Shaoxing University. This study was conducted in accordance with human subject protection regulations. This survey adopts stratified sampling, and the subjects of the survey are mainly freshmen, sophomores and junior students. According to the requirements of representativeness in the selection of questionnaire samples, and considering the popularization of research conclusions, we have reflected the characteristics of different types of schools and different majors when sampling. A total of 3410 questionnaires were distributed, 3401 were recovered and 3395 were valid.

### 2.1 Number of voluntary activities on campus

From the number of volunteer activities on campus, it can be seen that as the number of activities increases, the number and proportion of students in any grade are decreasing, and lower grade students are more likely to participate in volunteer activities. As shown in Table 1, the number of people in category A 1–5 accounted for the highest proportion, among which 991 were in freshman category A, accounting for 65.46%, 596 in sophomore category A, accounting for 54.78%, 404 in junior category A, accounting for 66.78%, and 120 in senior category A, accounting for 63.83%.

**Table 1:** Number of voluntary activities on campus.

		freshman		sophomore		junior		senior	
	Activity times	number	proportion	number	proportion	number	proportion	number	proportion
On Campus	A.1-5 Times	991	65.46%	596	54.78%	404	66.78%	120	63.83%
	B.6-10 Times	385	25.43%	313	28.77%	153	25.29%	39	20.74%
	C.11-20 Times	104	6.87%	113	10.39%	31	5.12%	15	7.98%
	D. More than 20 times	34	2.25%	66	6.07%	17	2.81%	14	7.45%
Off Campus	A.1-5 Times	490	85.96%	481	81.53%	298	86.38%	111	86.72%
	B.6-10 Times	56	9.82%	69	11.69%	32	9.28%	10	7.81%
	C.11-20 Times	15	2.63%	26	4.41%	11	3.19%	4	3.13%
	D. More than 20 times	9	1.58%	14	2.37%	4	1.16%	3	2.34%

The number of categories B with 6–10 times accounted for the second place, including 385 in category B, accounting for 25.43%, 313 in sophomore B, accounting for 28.77%, 153 in junior B, accounting for 25.29%, and 39 in senior B, accounting for 20.74%.

The number of class C with 11–20 times accounted for the third place, including 104 in major C, accounting for 6.87%, 113 in sophomore C, accounting for 10.39%, 31 in junior B, accounting for 5.12%, and 15 in senior B, accounting for 7.98%.

The number of people with more than 20 times in category D accounted for the fourth place, including 34 in freshman D, accounting for 2.25%, 66 in sophomore D, accounting for 10.39%, 17 in junior D, accounting for 2.81%, and 14 in senior D, accounting for 7.45%.

In addition, different grades in different categories of the proportion, also have different. In category A, the highest proportion was in the junior year, with 66.78%, followed by the freshman year, senior year and sophomore year. In category B, the highest proportion was in the sophomore year, with 28.77%, followed by the freshman year, junior year and senior year. In category C, the highest proportion was sophomore, 10.39%, followed by senior, freshman and junior. In category D, the highest proportion was in the senior year, with 7.45%, followed by the sophomore year, junior year and freshman year.

### 2.2 Number of voluntary activities outside the school

As can be seen from Table 1, the number of off-campus volunteer activities, with the increase of the number of activities, the number of people and their proportion are decreasing. At the same time, compared with campus activities, both the number and the proportion of participants have decreased compared with campus activities.

The number of people in category A 1–5 times accounted for the highest proportion, among which 490 were in freshman A, accounting for 85.96%, 481 in sophomore A, accounting for 81.53%, 298 in junior A, accounting for 86.38%, and 111 in senior A, accounting for 86.72%.

The number of class B 6–10 times accounted for the second place, including 56 in category B, accounting for 9.82%, 69 in sophomore B, accounting for 11.69%, 32 in junior B, accounting for 9.28%, and 10 in senior B, accounting for 7.81%.

The number of categories C 11–20 accounted for the third place, including 15 in major C, accounting for 2.63%, 26 in sophomore C, accounting for 4.41%, 11 in junior C, accounting for 3.19%, and 4 in senior C, accounting for 3.13%.

The number of people with more than 20 times in category D accounted for the fourth place, including 9 in freshman D, accounting for 1.58%, 14 in sophomore D, accounting for 2.37%, 4 in junior D, accounting for 1.16%, and 3 in senior D, accounting for 2.34%.

In addition, different grades in different categories of the proportion, also have different. In category A, the highest proportion was in the senior year, with 86.72%, followed by the freshman, sophomore and junior year. In category B, the highest proportion was sophomore, 11.69%, followed by freshman, junior and senior year. In category C, the highest proportion was sophomore, 4.41%, followed by junior, senior and freshman. In category D, the highest proportion is sophomore, 2.37%, followed by senior, freshman and junior.

### 2.3 Number of voluntary activities outside the school

As shown in Table 2, the main work place is A.community,C.voluntary agency.

The main place of freshman volunteer activities is A.community C. Volunteer institutions, and other locations are mainly concentrated in hospitals, courts, marathons, and crossing horses.

**Table 2:** Number of voluntary work place outside the school.

	freshman		sophomore		junior		senior	
1	A.community	146	A.community	157	A.community	103	A.community	103
2	C.voluntary agency	99	A.community C.voluntary agency	89	C.voluntary agency	52	C.voluntary agency	52
3	A.community C.voluntary agency	58	A.community B.village committee	74	A.community C.voluntary agency	49	A.community C.voluntary agency	49
4	A.community B.village committee C.voluntary agency	41	C.voluntary agency	73	A.community B.village committee	35	A.community B.village committee	35
5	A.community B.village committee	37	A.community B.village committee C.voluntary agency	60	A.community B.village committee C.voluntary agency	20	A.community B.village committee C.voluntary agency	20
6	D. Other hospitals	16	B.village committee	26	B.village committee	15	B.village committee	15
7	B.village committee	13	D. Other hospitals	17	D. Other hospitals	9	D. Other hospitals	9
8	D. Other courts	10	B.village committee C.voluntary agency	7	B.village committee C.voluntary agency	7	B.village committee C.voluntary agency	7
9	D. Other marathons"	10	D. Other schools	5	D. Other libraries	5	D. Other libraries	5
10	D. Other "Yue Ma"	10	A.community D. Other hospitals	4	A.community D. Other libraries	3	A.community D. Other libraries	3

The main place of the sophomore volunteer activities is A.community,B.village committee C. Volunteer institutions, and other locations are mainly concentrated in hospitals and schools.

The main place of the junior volunteer activities is A.community C. Voluntary institutions, and other locations are mainly concentrated in hospitals, voluntary institutions, and libraries.

The main location of senior volunteer activities is A.community C. Volunteer institutions, other places are mainly concentrated in the village committee, hospitals, libraries.

Therefore, it can be concluded that for the focus of different grades, the location of external voluntary activities is quite different. The location of the junior and senior years is focused on the library because of the influence of the postgraduate entrance examination or the civil servant examination.

### 2.4 What kind of volunteer activities to do outside the school

As shown in Table 3, volunteer service projects mainly include social community service, school service, hospital service, environmental protection service, civilization communication, care service, competition service; family services for parents and relatives; volunteer services for all departments on campus; industrial legal services, professional services.

**Table 3:** What kind of volunteer activities to do outside the school.

	freshman		sophomore		junior		senior	
1	A.accompany elderly people	60	B.nucleic acid testing	85	B.nucleic acid testing	48	B.nucleic acid testing	22
2	I.garbage classification	24	A.accompany elderly people	29	A.accompany elderly people	20	A.accompany elderly people	8
3	C. Theoretical preaching	24	A.accompany elderly people B.nucleic acid testing	26	C. Theoretical preaching	15	A.accompany elderly people B.nucleic acid testing	5
4	A.accompany elderly people I.garbage classification	21	C. Theoretical preaching	25	F. Safe patrol	14	I.garbage classification	4
5	F. Safe patrol	20	F. Safe patrol	21	A.accompany elderly people B.nucleic acid testing	13	G. Help "double reduction"	3
6	B.nucleic acid testing	19	B.nucleic acid testing I.garbage classification	17	B.nucleic acid testing C. Theoretical preaching	9	F. Safe patrol	3
7	A.accompany elderly people B.nucleic acid testing	18	B.nucleic acid testing F. Safe patrol	11	B.nucleic acid testing I.garbage classification	9	J. Other "Yue Ma"	2
8	D. Sunshine assistive	15	E.ecological construction	9	E.ecological construction	8	D. Sunshine assistive	2
9	J. Other marathons"	13	H.a program under which officials	9	G. Help "double reduction"	7	J. Other family tutors	2
10	J. Other marathon volunteers"	12	E.ecological construction I.garbage classification	9	I.garbage classification	7	A.accompany elderly people D. Sunshine assistive	2

According to the analysis, we can see that volunteering in the first to senior years in the main content of A.accompany elderly people, B.nucleic acid testing, C. Theory preaching, I.garbage classification.

The type of freshman volunteer activity is A.accompany elderly people, C. Theory preaching, I.garbage classification,. Other activities mainly focus on safety patrol, sunshine to help the disabled, marathon.

The main place of the sophomore volunteer activities is A.accompany elderly people, B. Nucleic acid testing, other sites mainly focus on theory propaganda, safety patrol, garbage classification, safety patrol, ecological construction, three to the countryside.

The main place of the junior volunteer activities is A.accompany elderly people, B.nucleic acid testing, C. Theory preaching, other places mainly focus on safety patrol, ecological construction, garbage classification, and help "double reduction".

The main location of senior volunteer activities is A.accompany elderly people, B. Nucleic acid testing, other places mainly focus on garbage classification, help "double reduction", safety patrol, sunshine to help the disabled, tutoring, yue horse.

It can be seen that college students participate in volunteer services in a wide range of different fields, including poverty alleviation and development, community construction, environmental protection, large-scale games, emergency rescue and disaster relief, foreign assistance. At the same time, the operation mechanism and organizational structure of college student volunteer organizations tend to be perfect, and a certain organizational culture has been formed.

### 2.5 Statistical analysis of the scores

Table 4 show the statistical analysis of the scores for Modern History of China (I), Ideological and moral cultivation and legal foundation, Introduction to MAO Zedong Thought (II) and the Theoretical System of Socialism with Chinese Characteristics (III).

**Table 4:** Test results of the outline of modern history of China.

	average	median	Standard deviation	Lower Quartile	Upper Quartile	Skewness	Kurtosis	Minimum	Maximum
I	80.55	81	7.99	75	86.25	-0.45	-0.15	59	100
II	82.41	83	8.45	79	89	-0.63	0.19	60	100
III	80.55	81	7.99	75	86.25	-0.45	-0.45	59	100

① central tendency

Median (81)> mean (80.54), which are basically the same.

② dispersion degree

The larger the variance and standard deviation, the more the data fluctuates.

③ distribution shape

The skewed coefficient (-0.452) is <0, so the data belongs to the left bias distribution. Because the value is between-0.5 and-0, it is considered as a moderately skewed distribution, and the deviation degree is not large.

It is generally believed that if a set of data is symmetrically distributed, the skewed coefficient is 0; if the skewed coefficient is greater than 1 or less than-1, it is highly skewed distribution, between 0,5–1 or-1 and-0,5, it is moderately skewed distribution. The closer the value is to 0, the lower the skewed degree will be.

The peak state coefficient (-0.149) <0, the data distribution is flat, the smaller the value, the more dispersed the data distribution. When greater than 0, the data are more concentrated, and flat when less than 0, the data distribution is more scattered.

### 2.6 Principal component analysis

**Fig 1:**
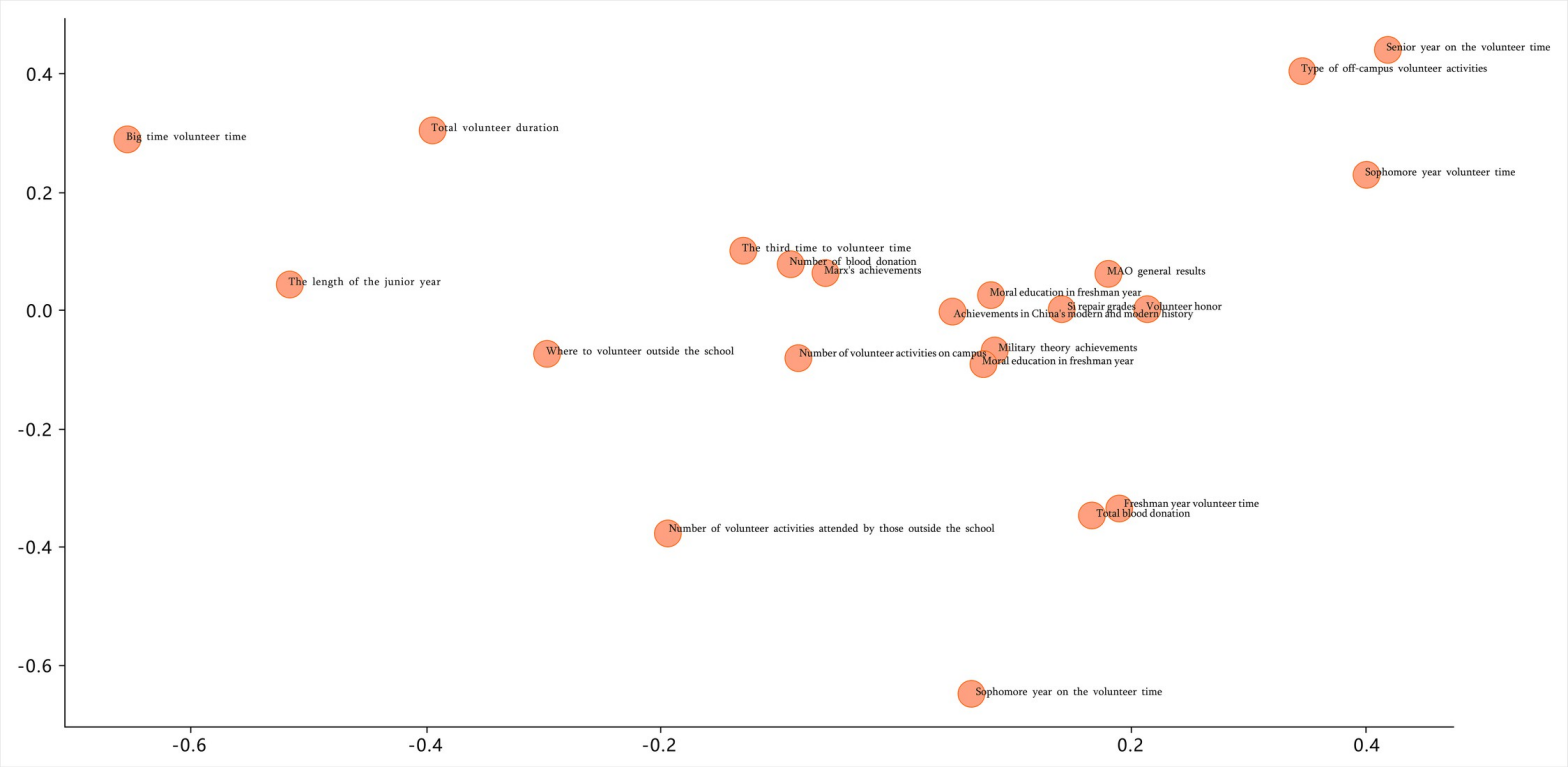
Two-dimensional analysis result.

**Fig 2:**
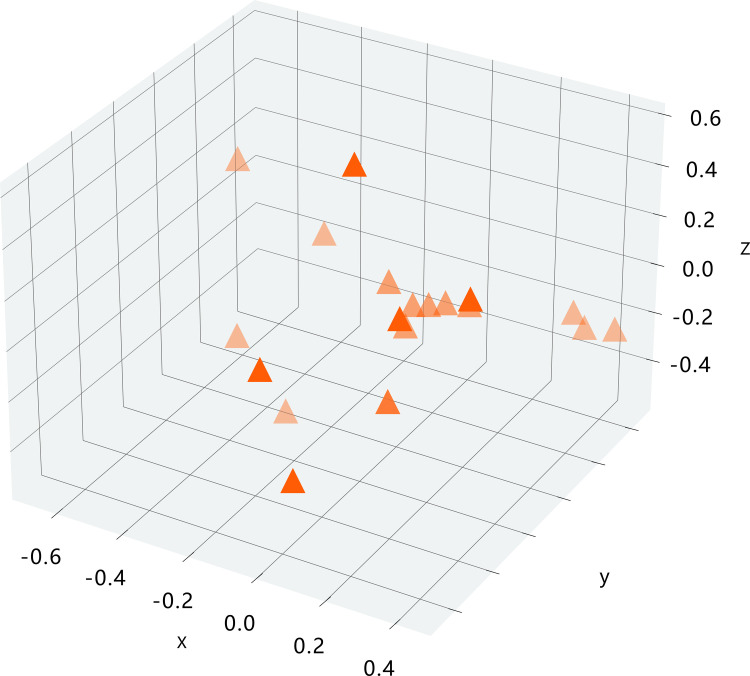
Three-dimensional analysis result.

Tables 5 and 6 show the data processed using principal component 2 D and 3 D analysis. And Figs 1 and 2 show the analysis results for the corresponding data.

**Table 5:** Two-dimensional analysis data.

**Number of volunteer activities on campus**	-0.08	-0.08
**Number of volunteer activities attended by those outside the school**	-0.19	-0.38
**Where to volunteer outside the school**	-0.30	-0.07
**Type of off-campus volunteer activities**	0.35	0.40
**Freshman year volunteer time**	0.19	-0.34
**Big time volunteer time**	-0.65	0.29
**Sophomore year on the volunteer time**	0.06	-0.65
**Sophomore year volunteer time**	0.40	0.23
**The length of the junior year**	-0.52	0.04
**The third time to volunteer time**	-0.13	0.10
**Senior year on the volunteer time**	0.42	0.44
**Total volunteer duration**	-0.39	0.30
**Volunteer honor**	0.21	-1.40
**Number of blood donation**	-0.09	0.08
**Total blood donation**	0.17	-0.35
**Moral education in freshman year**	0.08	-0.09
**Moral education in freshman year**	0.08	0.02
**Achievements in China’s modern and modern history**	0.05	-0.00
**Si repair grades**	0.14	0.00
**MAO general results**	0.18	0.06
**Marx’s achievements**	-0.06	0.06
**Military theory achievements**	0.08	-0.07

**Table 6:** Three-dimensional analysis data.

**Number of volunteer activities on campus**	-0.08	-0.08	0.56
**Number of volunteer activities attended by those outside the school**	-0.19	-0.38	-0.10
**Where to volunteer outside the school**	-0.30	-0.07	-0.53
**Type of off-campus volunteer activities**	0.35	0.40	-0.24
**Freshman year volunteer time**	0.19	-0.34	0.23
**Big time volunteer time**	-0.65	0.29	0.15
**Sophomore year on the volunteer time**	0.06	-0.65	-0.24
**Sophomore year volunteer time**	0.40	0.23	-0.04
**The length of the junior year**	-0.52	0.04	-0.38
**The third time to volunteer time**	-0.13	0.10	0.36
**Senior year on the volunteer time**	0.42	0.44	-0.25
**Total volunteer duration**	-0.40	0.30	-0.07
**Volunteer honor**	0.21	-1.40	0.08
**Number of blood donation**	-0.09	0.08	0.43
**Total blood donation**	0.17	-0.35	-0.10
**Moral education in freshman year**	0.08	-0.09	-0.01
**Moral education in freshman year**	0.08	0.02	0.00
**Achievements in China’s modern and modern history**	0.05	-0.00	0.01
**Si repair grades**	0.14	0.00	0.04
**MAO general results**	0.18	0.06	0.01
**Marx’s achievements**	-0.06	0.06	0.01
**Military theory achievements**	0.08	-0.07	0.06

### 2.7 The similarity calculation of voluntary attributes

In the system, it is necessary to obtain student attribute data and isomorphize reasonable representation function to construct student Embedding, and then calculate the similarity between students volunteer data as shown in Table 7.

**Table 7:** The similarity of attribute values for student A and B.

	A	B
1. Gender	woman	woman
2. College	School of Civil Engineering	art
3. Grade	senior	senior
4. Have you ever participated in any volunteer activities on campus?	yes	yes
5. How many volunteer activities did you participate in in each school year?	C.11-20 Times	B.6-10 Times
6. Have you ever participated in off-campus volunteer activities?	yes	yes
7. The number of voluntary activities attended outside the school in each academic year?	B.6-10 Times	B.6-10 Times
8. Where to do my volunteer activities outside the school?	C.voluntary agency	A.community
9. What kind of volunteer activities do you do outside the school?	B.nucleic acid testing	B.nucleic acid testing
13. Volunteer length of each semester since enrollment- -how total in the first semester of freshman year?	0	20
13. In the second semester of the freshman year, how much volunteer time is total?	45.16	20
The first semester of the sophomore year, how much volunteer time?	7.99	20
The second semester of the second year, how much volunteer time?	2.38	20
13. In the first semester of the junior year?	1.54	20
13. In the second semester of the third year?	3.56	23
In the first semester of the senior year, how much volunteer time is total?	40.14	17
15. How many volunteer hours in total since enrollment?	100.77	123
16. Volunteer honors won?	not have	Excellent volunteer of the hospital
17. How many blood donations since entering the school?	B.1-5 Times	B.1-5 Times
18. Total amount of blood donation since enrollment?	B.200cc~600cc	C.600cc~1000cc
Moral education in each semester since enrollment- -on the freshman year	ample	good people
Freshman	ample	good people
Sophomore	ample	ample
Sophomore	ample	ample
Junior year	ample	ample
23, results (no results, no fill)—"Outline of Modern History of China" exam results	77	88
"Ideological and moral cultivation and legal foundation" examination results	84	89
Introduction to MAO Zedong Thought and the Theoretical System of Socialism with Chinese Characteristics, examination results	76	86
"Marxist Principles" test results	83	93
23. The Military Theory test results	85.5	80

First of all, it is necessary to build the information matrix of known student information in the system. This table is the attribute information of student A. By calculating the similarity of the attribute information with the attribute information matrix of all students in the system, the student information B with the highest similarity is selected, and the moral education score of A is predicted according to the moral education score of the student B. Combine numerical calculation and text calculation to predict students’ moral education performance.

The Table 7 is an example to calculate the similarity of student A and B attribute values, including text similarity calculation and numerical similarity calculation. The similarity of text data, according to whether the item of A student is consistent with the corresponding item of A line in the matrix, the similarity is 1, and the similarity is 0. Numerical similarity is calculated based on jkard similarity coefficient, cosine similarity, Euclidean distance, Manhattan distance. Using similarity calculation, according to the classification and attribute value of the students with known moral education scores, the attribute value is known, but the students with moral education scores conduct similarity evaluation, and select the one with the highest similarity score, so as to find the data most similar to the students. Then the student’s moral education achievement evaluation is the closest.

### 2.8 Analysis of volunteer motivation and mental health

Volunteer service motivation refers to the psychological process and behavioral motivation of volunteers to participate in volunteer service. Volunteer motivation enables individuals to maintain their enthusiasm and volunteerism in different settings, with a strong positive relationship with volunteer behavior and sustainability. Self-determination theory holds that volunteer motivation is a self-determined intrinsic motivation that is closely related to psychological outcomes. Academic self-efficacy refers to the subjective judgment and evaluation of an individual on whether he can successfully complete academic tasks in the learning environment, and is the embodiment of self-confidence in the learning task. Research shows that this satisfaction can improve self-efficacy and confidence levels when students can meet their motivation for volunteering. As shown in Table 8, there is a good correlation between volunteering and mental health.

**Table 8:** Analysis of volunteer motivation and mental health.

Item	minimum value	maximum value
mental health	0	22
self-affirmation	0	11
melancholy	0	10
anxious	0	9
Volunteer service motivation	51	91
Learn to understand	5	19
vocational development	5	19
Value expression	5	17
ego trip	2	18
ego trip	4	18
self belay	5	17
social communication	4	17
Academic self-efficacy	46	111
Learning ability and self-efficacy	24	60
Learning behavior and self-efficacy	19	58

The motivation, learning understanding, career development, value expression, self-improvement, self-protection, and the highest self-efficacy, self-efficacy, self-affirmation factor, depression factor and anxiety factor.

As shown in Table 9, the total score of volunteer motivation and the score of each dimension were significantly positively correlated with the total score of mental health and self-affirmation factor, while the total score of volunteer motivation, learning understanding, career development, self-protection, and social interaction were significantly negatively correlated with the score of depression factor. The total score of volunteer motivation and the score of each dimension were significantly positively correlated with the total score of academic self-efficacy and the score of each dimension. The total score of academic self-efficacy and the scores of all dimensions were significantly positively correlated with the total score of mental health and self-affirmation factor, the total score of academic self-efficacy was negatively correlated with the factor of depression and anxiety, and the scores of learning self-efficacy and anxiety.

**Table 9:** The total score of volunteer motivation and the score of each dimension.

Item	mental health	self-affirmation	melancholy	anxious	Academic self-efficacy	Learning ability and self-efficacy	Learning behavior and self-efficacy
Volunteer service motivation	0.2044	0.2805	-0.1579	-0.0929	0.5367	0.5057	0.483
Learn to understand	0.2135	0.2075	-0.2332	-0.1108	0.4660	0.4111	0.4449
vocational development	0.2464	0.1824	-0.1910	-0.1551	0.4653	0.3825	0.4017
Value expression	0.1461	0.1728	-0.1402	-0.0563	0.3665	0.3987	0.297241959
ego trip	0.2221	0.2137	-0.1081	-0.0462	0.4922	0.4370	0.392292544
self belay	0.2291	0.2709	-0.2190	-0.1584	0.3591	0.4130	0.419
social communication	0.1855	0.1965	-0.1694	-0.1790	0.5094	0.4735	0.5309
Academic self-efficacy	0.4173	0.4950	-0.2028	-0.2049	1.0000		
Learning ability and self-efficacy	0.3986	0.4782	-0.1726	-0.1465	one	1.0000	
Learning behavior and self-efficacy	0.3576	0.4440	-0.1074	-0.1257		one	1.000

Volunteers want to learn more or show skills that are not often used in their volunteering process. The mental health of college student volunteers is in a good state, the level of self-affirmation is high, and the level of depression and anxiety is low. For mental health, learning from the actions or experience of volunteering can provide greater psychological security for volunteers.

Volunteer service is often associated with enhanced mental health and reduced depressive symptoms. Volunteering is likely to be therapeutic, and the volunteers develop positive influences through psychological growth. The total score of academic self-efficacy and the score of all dimensions were significantly and positively correlated with the total score of mental health and self-affirmation factor, and the total score of academic self-efficacy was negatively correlated with the score of depression and anxiety factors. This may be related to the connotation of self-efficacy, which underlies individual cognitive approaches, and can promote self-regulation and self-correction when managing stressors. Studies have shown that students with strong academic self-efficacy are able to persist to challenges and tend to have better mental health.

Although mental health cannot directly affect the volunteer motivation, mental health can indirectly affect the volunteer motivation through the mediation role of academic self-efficacy, and academic self-efficacy plays a complete mediating role in the relationship between mental health and volunteer motivation. Although there was a significant positive correlation between mental health and volunteering motivation, the structural equation model revealed that mental health is not directly related with volunteering motivation in the presence of academic self-efficacy. Although the mental health status of college students’ volunteers is related to the volunteer service motivation, college students are influenced by their academic self-efficacy, and college students with high volunteer enthusiasm will also feel forced to volunteer. Academic self-efficacy mainly reflects the academic situation of college students. When college students have great academic pressure, especially when facing the final exam, they will be slack, tired, and even want to withdraw from the psychology of volunteer service.

## 3. Discussion and conclusion

Through the above analysis and research, it is found that the status quo and existing problems of college students participating in voluntary activities are as follows.

In terms of the cognitive intention to participate in volunteer activities, the lower grade students are more enthusiastic to participate, and the scope of activities is concentrated in the school. Specifically speaking, first, a large number of college students participate in voluntary activities, but the gender difference is large. Girls were slightly more motivated to participate in the activities. Second, volunteer activities are mainly concentrated on campus and pay not enough attention to off-campus activities. Third, senior students still have the willingness to continue participating in volunteer activities.

In terms of the basic status of voluntary activities, the frequency of participation is low and the duration is short, the way of participation is single, the type structure needs to be optimized, and the purpose is mainly altruism. Specifically, first, the number of participation in voluntary activities is low, and concentrated in the form of short-term voluntary activities. Data show that the majority of the students who have participated in the volunteer activities are 1–5 times, and some students may be because of the conflict with their professional study time. Most students participated in less than 20 hours each time, which indicates that the activity duration is mostly short-term. Second, there is a single way to participate in volunteer activities. Most students participate in volunteer activities through schools or associations, and rarely participate through other ways, especially themselves. Third, the types of volunteer activities involved are relatively rich, but the structure needs to be further optimized. Statistics show that among the various types of volunteer activities, the most important events are on-campus environmental protection services, community or welfare home helpers for the elderly and the disabled, and sports meetings or marathons. This also reflects that the current organized activities are mainly the type of public welfare and love offering, and there are fewer activities that can practice the knowledge and skills learned.

### 3.1 Increase the length of volunteer service

The second class credits of learning, cognition and perception in the daily social practice volunteer service activities can be transformed into appropriate first class credits to encourage college students to complete the volunteer service of relevant required class hours within a specific time [6]. At the same time, through the volunteer service practice courses, network courses content of learning, college students combined with their own expertise, personal time, service category, practice credits consider required volunteer service online direction of the network and practical courses. This is not only conducive to the cultivation of college students’ ideological and moral cultivation and service concept cognition, but also can improve the employment level of college students and the adaptability of service positions. universitys and universities can give different credits according to college students’ participation time, activity level and activity effect, which can be included in the length of volunteer service. When college students participate in volunteer service activities of different content, different levels and different times, they can be recognized and evaluated by different credits [7].

### 3.2 Enrich volunteer services

Keeping up with the trend of The Times, keeping close to the key words of The Times and the concept of development, and constantly injecting the new vitality and new strength in the content of ideological and political education into the volunteer service of college students, which is helpful to create the conditions for the practical education, development and innovation of volunteer service. One is to increase the content of volunteer services that meet the needs of students and the actual needs of society. Second, make maximum use of social resources to enrich the content of college students’ volunteer services. In economic and social development, meet the growing social actual demand, colleges and universities should be closely linked with the resident communist youth league organizations, volunteers association, grassroots community, grasp the current trend of The Times, makes the university volunteer service and social demand trend together, so as to realize the balance between supply and demand, not only can promote social coordinated development, but also can enrich the content of college students’ volunteer service [8]. Colleges and universities should take the initiative to integrate the integration of college students’ volunteer service organization with the society, integrate and maximize the use of all resource advantages with the highest efficiency, and appropriately form a state of complementary advantages [9], so as to form a benign interaction with the ideological and political education of college students in the circular linkage inside and outside the school.

### 3.3 Establish a volunteer service network sharing platform

Network as the present college students popular convenient and effective, extensive use of communication platform, the actual development of volunteer service and college students, combining college students volunteers, service object, social resources, make volunteer service generally into college students’ daily life, constantly build college students to accept participation, set ideological education, information release, volunteer recruitment, course training, interactive communication, effective show and certification evaluation effect in the integration of college students volunteer service network sharing platform, namely the network ideological and political education center. On the volunteer service network sharing platform, college students can understand the current ideological value orientation, experience the information sharing of volunteer service [10], actively participate in the volunteer service registration according to their own status, and can also share the volunteer service exchange experience with students who have participated in the same volunteer service. Relying on the volunteer service network sharing platform, students participate in the record and evaluation of volunteer service activities, and realize the integration of ideological and political practice education that promotes the improvement of ideological and moral character of college students and the growth of volunteer service ability.

## Supporting information

S1 Data set (RAR)

S1 File (DOCX)
