# Supplementary material for: The impact of volunteer service on moral education performance and mental health of college students
Source: PLoS One. 2024 Apr 16;19(4):e0294586. doi: 10.1371/journal.pone.0294586 (PMC11020720; doi:10.1371/journal.pone.0294586)
Supplement: S1 File — (DOCX) [file pone.0294586.s002.docx]

**college students' Participation in Volunteer Service**

*Please confirm: this questionnaire is voluntary. After filling out, it is deemed that the information in it can be used for research on the impact of college students’ voluntary service on moral education performance and mental health.

1. Gender: male, female
2. College:
3. Grades: freshman, sophomore, junior, senior, and fifth
4. Have you participated in any volunteer activities on campus: yes, no
5. The number of volunteer activities participated in each school year:

1-5 times, 6-10 times, 11-20 times, more than 20 times

1. Have you ever participated in off-campus volunteer activities: Yes, No
2. The number of volunteer activities outside the school each academic year:

1-5 times, 6-10 times, 11-20 times, more than 20 times

1. Where do you do volunteer activities outside of school (multiple choice):

community, village committee, support organization, others

1. What kind of voluntary activities do you do outside of school (multiple choice):

A. Accompanying the elderly

B. Nucleic acid detection

C. Theoretical presentation

D. Sunshine helps the disabled

E. Ecological construction

F. Safety Patrol

G. Assist in "double reduction"

H. Going to the countryside three times

I. Garbage Sorting

J. Others

10. Volunteer hours per semester since enrollment

In the first semester of freshman year, how many volunteer hours in total:

In the second semester of freshmen, how many volunteer hours in total:

In the first semester of the sophomore year, how many volunteer hours in total:

In the second semester of the sophomore year, how many volunteer hours in total:

In the first semester of junior year, how many volunteer hours in total:

In the second semester of junior year, how many volunteer hours in total:

In the first semester of senior year, how many volunteer hours in total:

In the second semester of senior year, how many volunteer hours in total:

In the first semester of the fifth year, how many volunteer hours in total:

11.Since entering school, how many hours have you volunteered in total:

12.What are the voluntary honors obtained?

13.Number of blood donations since enrollment: 0 times, 1-5 times, 6-10 times, more than 10 times

14.The total amount of blood donation since enrollment:

<200cc, 200cc-600cc, 600cc-1000cc, more than 1000cc

15. Moral education in each semester since enrollment:

Freshman year: excellent, good, pass

Major: Excellent, Good, Pass

Sophomore Upper: Excellent, Good, Pass

Sophomore Lower: Excellent, Good, Pass

Junior High: Excellent, Good, Pass

Junior Lower: Excellent, Good, Pass

Senior Senior: Excellent, Good, Pass

Senior year: Excellent, Good, Pass

16. Grades (if you don't have one, fill in none)

"Outline of Modern Chinese History" Exam Results

"Ideological and Moral Cultivation and Legal Basis" Exam Results

"Introduction to Mao Zedong Thought and Introduction to the Theoretical System of Socialism with Chinese Characteristics" Examination Results

"Principles of Marxism" Exam Results

"Military Theory" Exam Results
